# A Preoperative MRI-Based Radiomics-Clinicopathological Classifier to Predict the Recurrence of Pituitary Macroadenoma Within 5 Years

**DOI:** 10.3389/fneur.2021.780628

**Published:** 2022-01-05

**Authors:** Yu Zhang, Yuqi Luo, Xin Kong, Tao Wan, Yunling Long, Jun Ma

**Affiliations:** ^1^Department of Radiology, Beijing Tiantan Hospital, Capital Medical University, Beijing, China; ^2^School of Biomedical Science and Medical Engineering, Beijing Advanced Innovation Centre for Biomedical Engineering, Beihang University, Beijing, China; ^3^Department of Biomedical Engineering, School of Biomedical Engineering, Capital Medical University, Beijing, China; ^4^Beijing Neurosurgical Institute, Beijing Tiantan Hospital, Beijing, China

**Keywords:** pituitary macroadenoma, recurrence, predictive model, deep learning, multilayer perceptron

## Abstract

**Objective:** To investigate the ability of a MRI-based radiomics-clinicopathological model to predict pituitary macroadenoma (PMA) recurrence within 5 years.

**Materials and Methods:** We recruited 74 recurrent and 94 non-recurrent subjects, following first surgery with 5-year follow-up data. Univariate and multivariate analyses were conducted to identify independent clinicopathological risk factors. Two independent and blinded neuroradiologists used 3D-Slicer software to manually delineate whole tumors using preoperative axial contrast-enhanced T1WI (CE-T1WI) images. 3D-Slicer was then used to extract radiomics features from segmented tumors. Dimensionality reduction was carried out by the least absolute shrinkage and selection operator (LASSO). Two multilayer perceptron (MLP) models were established, including independent clinicopathological risk factors (Model 1) and a combination of screened radiomics features and independent clinicopathological markers (Model 2). The predictive performance of these models was evaluated by receiver operator characteristic (ROC) curve analysis.

**Results:** In total, 1,130 features were identified, and 4 of these were selected by LASSO. In the test set, the area under the curve (AUC) of Model 2 was superior to Model 1 {0.783, [95% confidence interval (CI): 0.718—.860] vs. 0.739, (95% CI: 0.665–0.818)}. Model 2 also yielded the higher accuracy (0.808 vs. 0.692), sensitivity (0.826 vs. 0.652), and specificity (0.793 vs. 0.724) than Model 1.

**Conclusions:** The integrated classifier was superior to a clinical classifier and may facilitate the prediction of individualized prognosis and therapy.

## Introduction

Pituitary adenoma is one of the most prevalent intracranial masses that can affect adults (1, 2). The varied clinical manifestations usually result from the endocrine activity, or volume of tumors. The classification of PA is based on different criteria, such as size, immunohistochemistry (IHC), invasion, hormone secretion, and clinical manifestation (1, 3). PAs are classified into micro, macro, and giant adenomas by the MRI size. The IHC subtypes of PAs are composed of growth hormone (GH), prolactin (PRL), adrenocorticotropic hormone (ACTH), thyroid-stimulating hormone (TSH), and follicle-stimulating hormone-luteinising hormone (FSH-LH), including the monohormonal and plurihormonal adenomas. Although benign in terms of their biological behavior, 30–45% of tumors invade the cavernous or sphenoid sinus, which can be categorized into invasive and non-invasive adenomas (1, 3, 4). According to the clinical classification, PAs consist of functioning and non-functioning types (1).

The treatment strategy for most tumors is operation. The postsurgical recurrence rate of pituitary macroadenoma (PMA) within 5 years is considerably high (5). The tendency to relapse has been related to many factors, including different histotypes, tumor remnants, or the extent of invasion into adjacent anatomical structures (6). Previous research has demonstrated that many clinicopathological prognostic tools have potential to predict the recurrence of PMA, consisting of IHC characteristics, invasion of tumors, genetic expression, and markers of proliferation (7–11). However, very few attempts have been made to integrate these risk factors with a machine-learning approach.

Radiomics is a form of analysis that quantitatively extracts imaging features from medical data (12). Thus far, radiomics studies of PA have predominantly focused on two aspects: presurgical evaluation and subtype classification (13–17). Some researchers have used radiomics to explore the potential for relapse in PA. However, these previous models have been associated with small sample sizes and only cases involving non-functioning tumors (18, 19).

In the present study, we aimed to establish a comprehensive classification model that combined independent clinicopathological risk factors with preoperative radiomics signatures for the prediction of PMA recurrence within 5 years of surgery. Our goal was to provide an efficient tool for guiding clinical management and predicting prognosis.

## Materials and Methods

### Ethics Statement

This retrospective study involved human subjects and was approved by the Ethics Committee of Beijing Tiantan Hospital. The requirement for written informed consent was waived.

### Subjects

The recurrence of adenomas was defined as incidence of enlarged remnant tumors in non-functioning PMAs, and/or endocrine biochemical recurrence in functioning PMAs (20, 21).

PMAs referred to adenomas with preoperative size > 10 mm by MRI in our study, based on Asioli et al. (20). PMAs were classified into immunonegative, monohormonal-including GH-positive, PRL-positive, ACTH-positive, FSH-LH-positive, TSH-positive, and plurihormonal by the results of IHC staining (22). The radiological signs of aggressive tumors were determined according to Knosp and Hardy—Wilson classifications on preoperative MRI by a blinded and experienced neuroradiologist. The Knosp and Hardy—Wilson criteria were used to evaluate the degree of invasion of cavernous sinus (CS) and suprasella, respectively. Knosp Scores 3 and 4 were described as adenomas extending beyond the lateral tangents of the cavernous segment of internal carotid artery (ICA) on coronal MRI and completely involving CS and ICA. Hardy—Wilson Grades 3 and 4 were represented as local and extensive invasion of the sellar floor; Stages C and D and E were characterized as total replacement of the third ventricle, intracranial adenomas, and invasion of CS (Supplementary Material 1) (5, 23). The aggressive PMAs were defined as grade of Knosp 3 or 4, and/or Hardy—Wilson Grades 3 or 4 (and/or Hardy—Wilson Stage C or D or E), and/or histological evidence of invasion of cavernous or sphenoid sinus (1). The patients who experienced subtotal resection were recognized as cases with residual tumor, and the subjects who underwent gross- or near-total resection were regarded as cases without remnants (24, 25).

A total of 168 consecutive postoperative subjects with a confirmed pathological diagnosis of PMA were acquired from our institutional medical database between January 2010 and December 2015. Analysis of medical records showed that 74 of these patients reported recurrent attacks (39 men/35 women); and 94 patients had not experienced recurrence (43 men/51 women). All the patients completed the 5-year follow-up period. The inclusion criteria were as follows: (1) available investigation for medical data; (2) underwent surgery; (3) had preoperative MRI; and (4) followed-up for duration of 5 years since first surgery. The exclusion criteria included (1) underwent other treatments for PMA before the first surgery or during the follow-up period; (2) pituitary apoplexy; (3) multiple intracranial lesions; and (4) poor-quality image or lack of contrast-enhancement MRI.

### MRI Acquisition and the Segmentation of Tumors

All enrolled subjects underwent MRI of the head prior to surgery, including several different acquisition protocols [axial T1WI and T2WI, axial, coronal, and sagittal contrast-enhanced T1WI (CE-T1WI)]. The contrast agent, dimeglumine gadopentetate, was injected at a dose of.2 ml/kg, following pre-contrast T1 scanning. MRI images were obtained from four different MRI scanners with 3 T (GE Discovery MR 750, *n* = 59; Siemens MAGNETOM Trio TimSystem, *n* = 43; Siemens MAGNETOM Verio, *n* = 22; Philips Ingenia, *n* = 9), and a 1.5 T scanner (GE Medical System Genesis Signa, *n* = 35). Supplementary Material 2 shows the type of the contrast medium and the parameters used for axial CE-T1WI for five MRI modalities.

Whole tumors, based on preoperative axial CE-T1WI images, were identified as the region of interest (ROI). The manual delineation of each ROI was conducted by a neuroradiologist with 5 years of experience, using 3D-Slicer software (version 4.10.2 r28257, National Institutes of Health). Prior to segmentation, we applied three steps to standardize different MRI images: N4ITK bias correction, resampling with resampled voxel sizes of 1, 1, and 1, and Laplacian of Gaussian (LOG) with LOG kernel sizes being 1.5, 2, and 2.5 by 3D-Slicer.

### Assessments of Intra- and Interobserver Reproducibility

Neuroradiologist 1 segmented the ROIs of 60 randomly selected cases on two occasions separated by an interval of 2 weeks. Neuroradiologist 2 with 5 years of experience independently performed the same analyses on one occasion. Intraclass correlation coefficient (ICC) was then calculated by R (version 4.0.2, http://www.R-project.org) to compare intra- and inter-observer reproducibility. The high reproducibility of these radiomics features was recognized as the ICC score for Radiologist 1 (on two occasions) or between Radiologists 1 and 2 >.75.

### The Extraction of Features and Dimension Reduction

In total, 1,130 features were extracted from the segmented ROIs by 3D-Slicer software. These features encompassed eight types: first-order, shape, gray-level dependence matrix (GLDM), gray-level co-occurrence matrix (GLCM), gray-level run length matrix (GLRLM), gray-level size zone matrix (GLSZM), neighboring gray tone difference matrix (NGTDM), and wavelet-based features, which were four distinct categories: intensity histogram, texture, shape, and wavelet. The detailed information for all features is shown in Supplementary Material 3.

The features with ICC score <0.75 were excluded in the first stage, because of the poor reproducibility. Then, we performed the least absolute shrinkage and selection operator (LASSO) in the R environment to carry out dimensionality reduction in the training set. The corresponding regularization coefficient (λ) was obtained by 10-fold cross-validation in LASSO regression based on the 1-standard error of the minimum criteria (1-SE criteria).

### The Establishment and Validation of a Radiomics-Clinicopathological Model

The z-score was used to normalize all features onto a similar scale. We randomly separated these subjects into a training set (including 51 recurrence and 65 non-recurrence subjects) and a test set (23 cases with relapse and 29 without relapse; based on a data-partition ratio of 7:3).

Two multilayer perceptron (MLP) classifiers were built by python (version 3.8.2, http://www.python.org) for the prediction of recurrence in PMA, including independent clinicopathological risk factors (Model 1—clinical model) and a combination of screened radiomics features and independent clinicopathological markers (Model 2—integrated model). Receiver operator characteristic (ROC) curves were performed and used to estimate the predictive performance of the two models by area under curve (AUC) analysis. This analysis allowed us to determine the accuracy, specificity, and sensitivity of each model.

**Figure 1:**
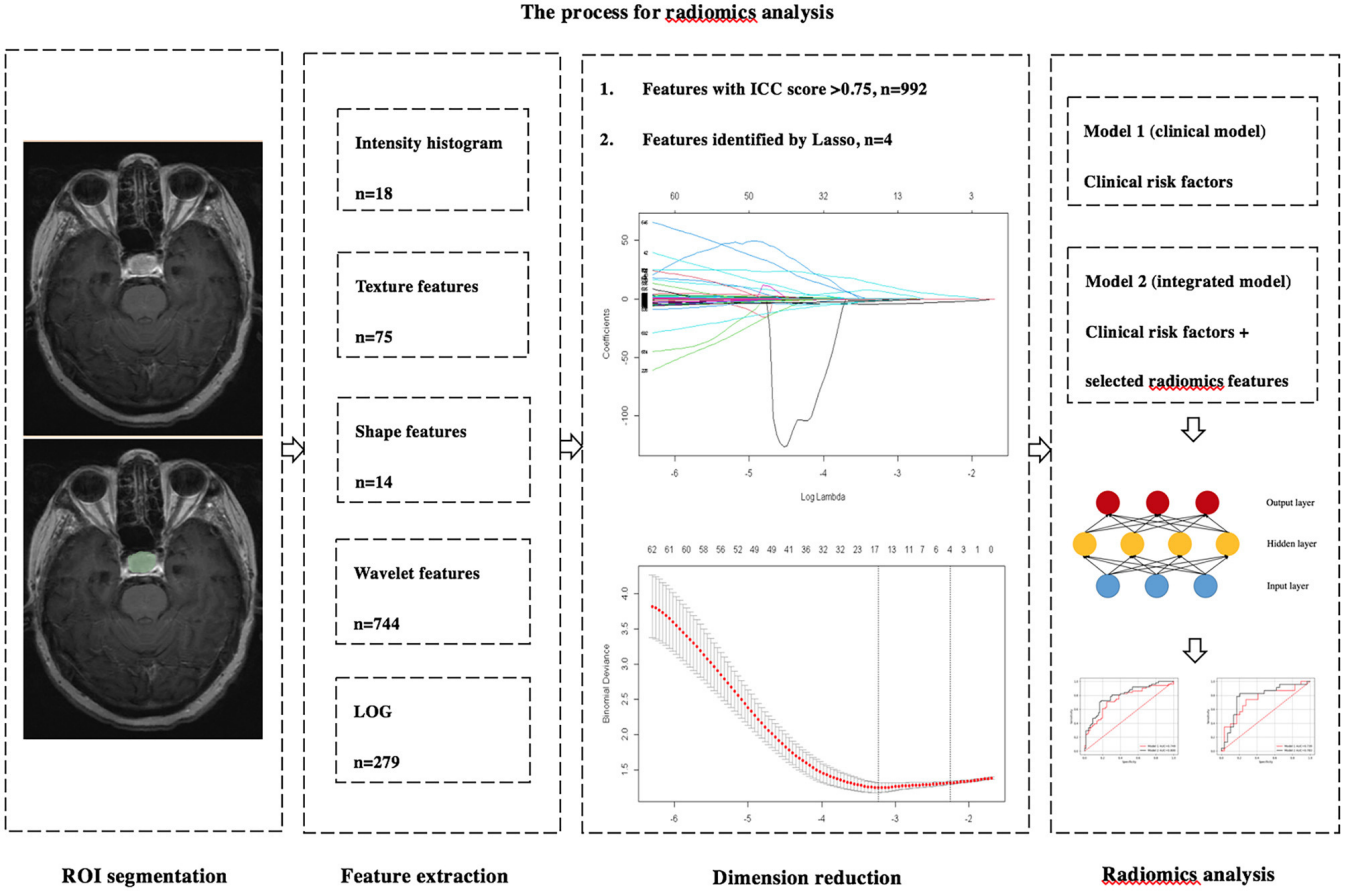
The process used for the analysis of radiomics. Radiomics features were extracted from the preoperative axial CE-T1WI images by 3D slicer. Dimension reductions were performed two times by ICC and LASSO. The MLP was used to build two predictive models. Model 1 included independent clinicopathological risk factors. Model 2 included the combination of radiomics features and independent risk factors.

MLP was composed of an input layer, a hidden layer, and an output layer. In the process of forward propagation, a series of algorithms were performed to obtain the output of each layer, which was used to be the input of the next layer. The equation was as follows:


$$\begin{array}{l} \text{y}=\text{f }\left(\text{wx}+\text{b}\right) \end{array}$$

where y represents the outcome of output, x represents the input vector, w represents the weight, b represents the bias, and f represents the activation function. Our classifiers included three hidden layers, for which the numbers of neurons were 64, 512, and 64, respectively. The dropout layer was conducted to lose 20% of neurons to reduce overfitting. We applied Rectified Linear Unit (ReLU) and Sigmoid to be activation functions for the hidden and output layers. The binary cross entropy was calculated for use as loss function. The weights were tuned by the back propagation method based on the derivation of the chain rule. In our study, the training epochs were set to 500. Before model establishment, the training cohort was shuffled. The monitoring indicators were accuracy, sensitivity, specificity, and AUC in the training set. Stochastic gradient descent (SGD) was used as the optimizer, with an initial learning rate of.1. The learning rate decay strategy was set to the reduction of 70% if the accuracy of training cohort did not improve for consecutive 100 epochs. Optimized class weights were obtained according to the numbers of recurrent and non-recurrent patients in the training set, and the batch size was default value of 32. The predictive performance of each model was validated in the test cohort and evaluated by 5-fold cross-validation. Figure 1 shows the process used for the analysis of radiomics.

### Statistical Analysis

The normality test of the data was performed by Shapiro—Wilk. Two-sided independent sample *t*-test and Mann—Whitney *U*-test were conducted to compare the differences in continuous variates, and Pearson's χ^2^ test and Fisher's precision probability test were used to investigate the differences in categorical variates in the training and test sets. Univariate and multivariable logistic regression were used to identify independent clinicopathological risk factors for the recurrence of PMA in the training set. The differences of extracted radiomics features between groups of recurrence and non-recurrence in the training set were determined by Mann—Whitney *U*-test; these analyses were carried out with SPSS (version 23.0, IBM), and a *p* of < 0.05 was considered to be statistically significant.

## Results

### Clinical Characteristics of the Study Cohort

The baseline investigation of the study patients is shown in Table 1. The differences with regard to clinical characteristics between training and test cohorts were not statistically significant.

**Table 1:** Clinical characteristics of PMA subjects in the training and test sets.

	**Training set** **(***n*** = 116)**	**Test set** **(***n*** = 52)**	**Whole set** **(***n*** = 168)**	***p***
**Age, mean (SD), y**	45.59 (12.38)	44.96 (11.63)	45.39 (12.12)	0.758^†^
**Sex, No. (%)**				0.899^§^
Male	57 (49.14)	25 (48.08)	82 (48.81)	
Female	59 (50.86)	27 (51.92)	86 (51.19)	
**Endocrine level, No. (%)**				0.214^§^
Non-functioning	71 (61.21)	37 (71.15)	108 (64.29)	
Functioning	45 (38.79)	15 (28.85)	60 (35.71)	
**Height, median (IQR), mm**	30.73 (20.42-41.04)	31.82 (19.21-44.43)	30.96 (25.81-36.93)	0.284^‡^
**Residual tumor, No. (%)**				0.584^§^
Without	43 (37.07)	17 (32.69)	60 (35.71)	
With	73 (62.93)	35 (67.31)	108 (64.29)	
**Invasion, No. (%)**				0.578^§^
No	33 (28.45)	17 (32.69)	50 (29.76)	
Yes	83 (71.55)	35 (67.31)	118 (70.24)	
**Surgical methods, No. (%)**				0.686^§^
Craniotomy	29 (25.00)	12 (23.08)	41 (24.40)	
Trans-sphenoidal	59 (50.86)	30 (57.69)	89 (52.98)	
Endoscopic	28 (24.14)	10 (19.23)	38 (22.62)	
**The IHC subtypes, No. (%)**				0.906^&^
Immunonegative	48 (41.38)	25 (48.08)	73 (43.45)	
GH-positive	6 (5.17)	1 (1.92)	7 (4.17)	
PRL-positive	5 (4.31)	3 (5.77)	8 (4.76)	
ACTH-positive	11 (9.48)	3 (5.77)	14 (8.33)	
FSH-LH-positive	22 (18.97)	8 (15.38)	30 (17.86)	
TSH-positive	2 (1.72)	1 (1.92)	3 (1.79)	
Plurihormonal	22 (18.97)	11 (21.15)	33 (19.64)	

*ACTH, adrenocorticotropic hormone; FSH, follicle-stimulating hormone; GH, growth hormone; IHC, immunohistochemistry; IQR, interquartile range; LH, luteinizing hormone; No, number; PRL, prolactin; SD, standard deviation; TSH, thyroid-stimulating hormone*.

† *Two-sided independent sample t-test*.

‡ *Mann—Whitney U-test*.

§ *Pearson's χ^2^ test*.

& *Fisher's precision probability test*.

Univariate analysis demonstrated that age {*p* = 0.034; OR, 0.967 [95% confidence interval (CI), 0.937–0.997]}, height [*p* = 0.007; OR, 1.068 (95% CI, 1.018–1.120)], residual tumor [*p* = 0.009; OR, 2.963 (95% CI, 1.319–6.659)], and invasion [*p* = 0.009; OR, 3.359 (95% CI, 1.359–8.305)] were significant risk factors for relapse. Multivariate analysis identified two independent risk factors for the recurrence of PMA: age [*p* = 0.035; OR, 0.963 (95% CI, 0.930–0.997)] and residual tumor [*p* = 0.047; OR, 2.393 (95% CI, 1.011–5.667)] (Table 2). Our study included four clinicopathological features: age, height, residual tumor, and invasion in Models 1 and 2 based on univariate and multivariate analyses.

**Table 2:** Univariate and multivariate analysis of clinical characteristics to identify risk factors in the recurrence of PMA in the training set.

	**Univariate analysis**		**Multivariate analysis**	
	**OR (95%CI)**	***p***	**OR (95%CI)**	***p***
**Age, y**	0.967 (0.937–0.997)	0.034^*^	0.963 (0.930–0.997)	0.035^*^
**Sex**				
Male (ref.)	1			
Female	1.008 (0.484–2.100)	0.982		
**Endocrine level**				
Non-functioning (ref.)	1			
Functioning	1.196 (0.564–2.535)	0.641		
Height, mm	1.068 (1.018–1.120)	0.007^*^	1.045 (0.992–1.100)	0.097
**Residual tumor**				
Without (ref.)	1			
With	2.963 (1.319–6.659)	0.009^*^	2.393 (1.011–5.667)	0.047^*^
**Invasion**				
No (ref.)	1			
Yes	3.359 (1.359–8.305)	0.009^*^	2.746 (0.994–7.585)	0.051
**Surgical methods**				
Transcranial (ref.)	1			
Trans-sphenoidal	0.904 (0.371–2.202)	0.824		
Endoscopic	0.595 (0.206–1.722)	0.338		
**The IHC subtypes**				
Immunonegative (ref.)	1			
Monohormonal	0.821 (0.358–1.881)	0.640		
Plurihormonal	2.450 (0.865–6.939)	0.092		

*CI, confidence interval; IHC, immunohistochemistry; OR, odds ratio*.

* *p < 0.05*.

### Intra- and Interobserver Analyses

The mean ICC scores for intra- (Neuroradiologist 1 on two occasions) and interobserver (Neuroradiologists 1 and 2) agreements were 0.913 ± 0.129 and 0.903 ± 0.127, respectively, for all selected patients, which showed the high agreement of these features.

### Comparing the Predictive Performance of the Two Models

**Figure 2:**
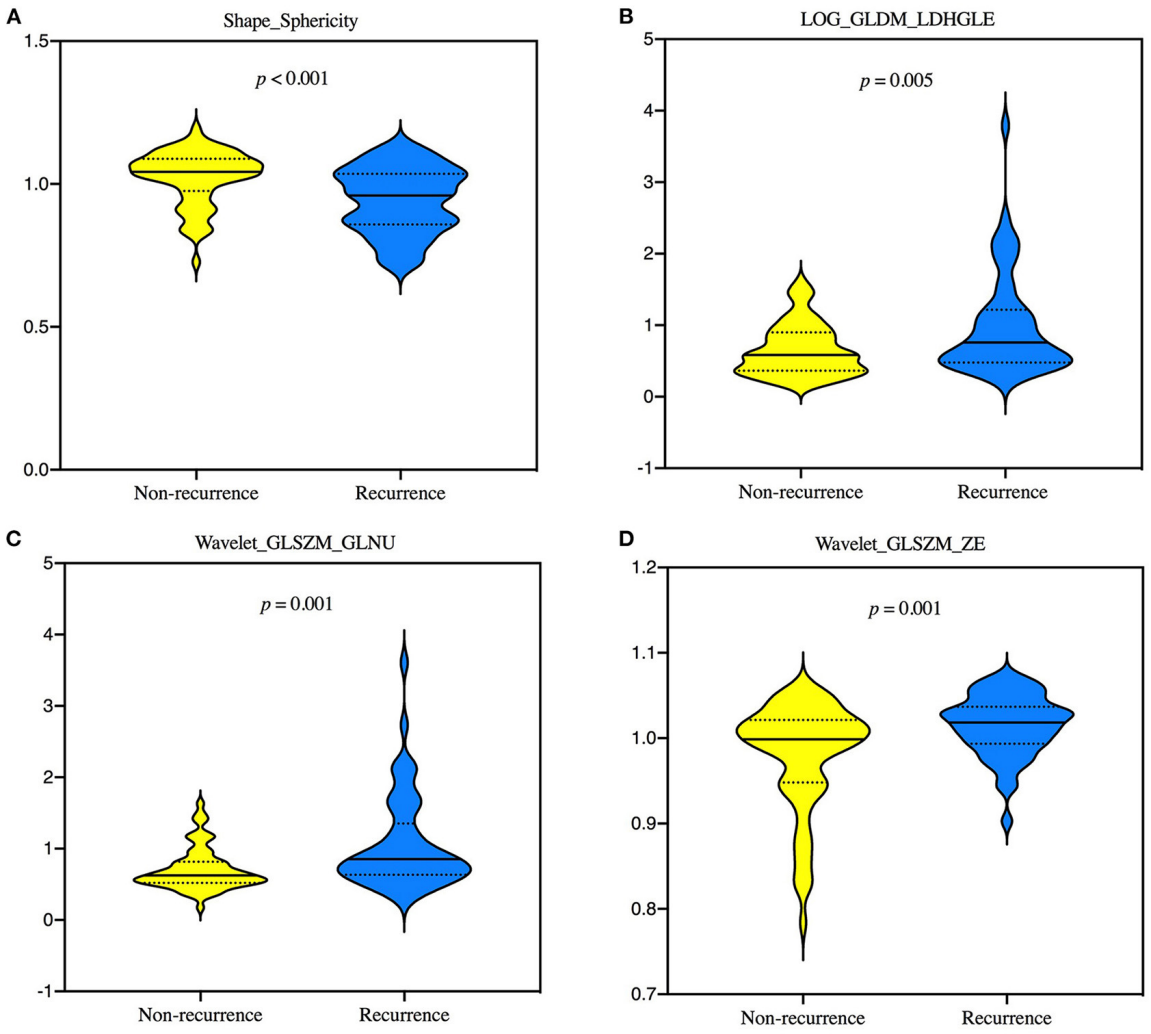
Violin plots showing the differences of 4 selected radiomics features, Shape_Sphericity **(A)**, LOG_GLDM_LDHGLE **(B)**, Wavelet_GLSZM_GLNU **(C)**, and Wavelet_GLSZM_ZE **(D)** between groups of recurrence and non-recurrence in the training set by Mann—Whitney *U*-test. GLDM, gray-level dependence matrix; GLNU, gray-level non-uniformity; GLSZM, gray-level size zone matrix; LDHGLE, large dependence high-gray-level emphasis; LOG, laplacian of gaussian; ZE, zone entropy.

Of the 1,130 features, 138 with unsatisfactory agreement were excluded by the first round, and four of these radiomics features were then identified by LASSO regression, consisting of one shape feature, one LOG, and two wavelet features. All of four selected signatures showed statistically significant differences (*p* < 0.05) in the training set (Figure 2).

**Figure 3:**
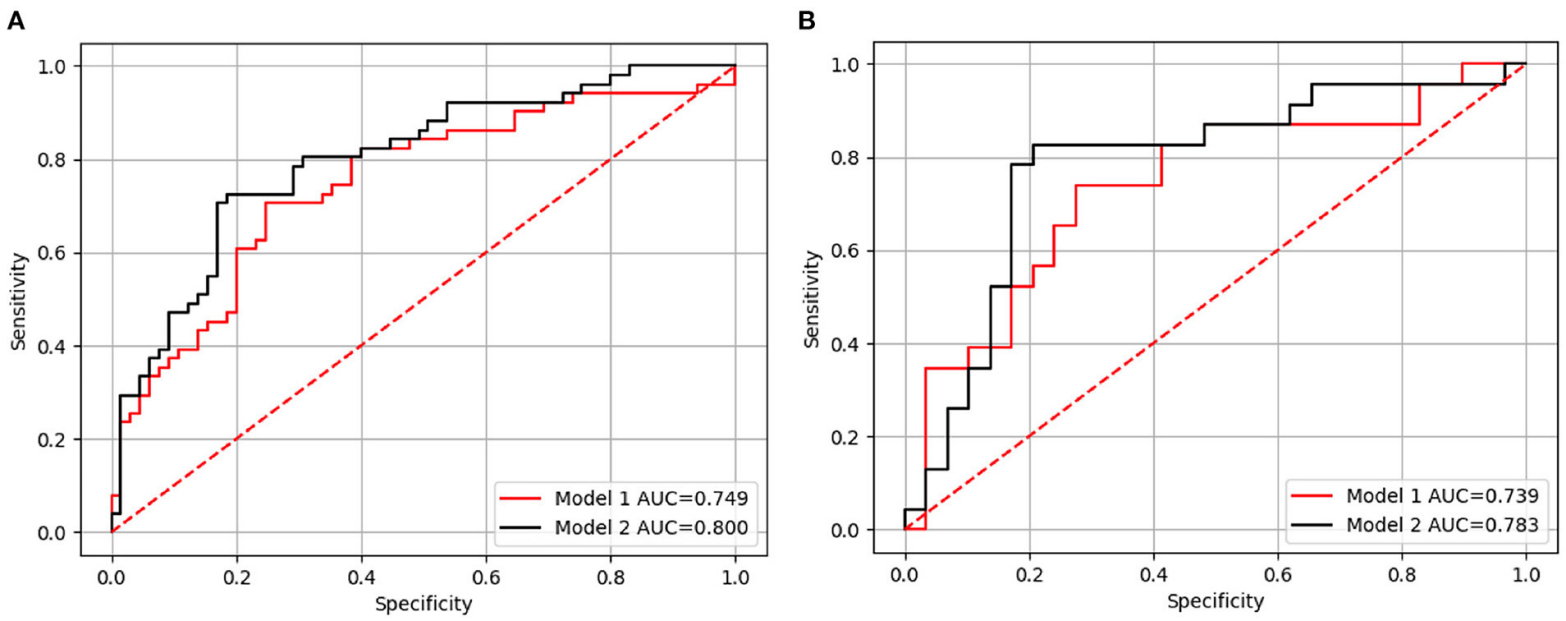
The receiver-operating characteristic (ROC) curve for Models 1 and 2 in the training **(A)** and test sets **(B)**, respectively. Model 1 included independent clinicopathological risk factors and Model 2 included both radiomics features and independent clinicopathological risk factors.

The ROC curves in the training and test sets are shown in Figure 3; the AUCs, accuracy, sensitivity, and specificity of the two models are presented in Table 3. In the test set, the AUC of Model 2 was superior to Model 1 [0.783, (95% CI: 0.718–0860) vs. 0.739, (95% CI: 0.665–0.818)]. Model 2 also yielded the higher accuracy (0.808 vs. 0.692), sensitivity (0.826 vs. 0.652), and specificity (0.793 vs. 0.724) than Model 1.

**Table 3:** Predictive performance of Models 1 and 2 in the training and test set.

	**Training set** **(***n*** = 116)**				**Test set** **(***n*** = 52)**			
	**AUC (95% CI)**	**ACC**	**SEN**	**SPE**	**AUC (95% CI)**	**ACC**	**SEN**	**SPE**
Model 1	0.749 (0.702–0.804)	0.724	0.706	0.738	0.739 (0.665–0.818)	0.692	0.652	0.724
Model 2	0.800 (0.759–0.845)	0.776	0.725	0.815	0.783 (0.718–0.860)	0.808	0.826	0.793

*Model 1 included independent clinicopathological markers and Model 2 included both radiomic features and clinicopathological markers*.

*AUC, area under the curve; ACC, accuracy; CI, confidence interval; SEN, sensitivity; SPE, specificity*.

## Discussion

In this study, we constructed a diagnostic classification strategy based on the presurgical MRI to predict the recurrence risk of PMA within 5 years. This comprehensive classifier incorporates clinicopathological and radiomics features and can accurately predict the recurrence of PMA.

Various factors are known to be associated with a higher risk of PMA recurrence, which remains a significant problem for both clinicians and patients. Very few previous reports have attempted to combine clinicopathology analysis with radiomics for the prediction of PMA. MRI radiomics approaches have been described in previous literature. For example, Zhang et al. (18) and Machado et al. (19) used this method to explore the recurrence of non-functioning PA. Compared with these previous results, the Model 2 in our study presented with relative low diagnostic accuracy. This is probably because we built the test set using an independent set of subjects rather than the training group. Moreover, our study simultaneously included functioning and non-functioning PMAs. The study cohorts and enrollment criteria may lead to the different predictive performances. However, our Model 2, which had a relatively large sample size and incorporated comprehensive markers, showed a better level of classification performance than Model 1. This improved predictive efficiency demonstrates that the combination of clinicopathological data and imaging may provide more practical information and guidance for developing a treatment and prognosis strategy than clinical analysis alone.

MLP model is a feed-forward artificial neural network (ANN) model that is applicable to a non-linear inseparable issue; Almubark et al. demonstrated the predictive value of this approach in their previous study (26). The generalization and efficacy of this method have been widely confirmed in several papers (27–29). Given these characteristics, we also established an MLP classifier for the recurrence of PMA and achieved satisfactory levels of predictive performance in a test cohort. These data indicate that this deep learning algorithm is a reproducible and robust technique for classification.

Many risk factors are associated with the recurrence of PMA. In the present study, we incorporated some of the primary predictors that have been described in previous literature (30). Four clinicopathological risk factors—age, height, invasion, and residual tumor—were finally included in our comprehensive model; these factors were identified by a combination of univariate and multivariate analyses. We found that the patients in the recurrence group tended to be younger. We believe that this is because there is a greater risk of gene disorders in younger age groups (31). In a previous study, Trott concluded that young patients express elevated levels of ki-67 in non-functioning pituitary adenoma, and that this is strongly associated with relapse (32). Moreover, some ultrastructural types of PMA resulted in regrowth, such as sparsely granulated somatotroph adenoma, and are more likely to affect younger patients. The aggressive growth pattern of PMA is one of the main reasons concerned with the prognosis (33). The invasive tumors usually exhibit more rapid growth, a higher proliferative index, and larger size. Thus, the severe erosion of surrounding structures (e.g., cavernous sinus and sellar floor) and great extension of the supra- and para-sellar lead to increased rates of recurrence (34). Similar results in the present literature provide support to our conclusion that tumor remnant is also known to be significantly correlated with PMA behavior, especially the higher incidence of larger extra-sellar residuals (5, 6, 34). Height is another recognized predictive candidate. It is evident that the tumors with higher height may result in incomplete resection and invasive behavior, which consequently raise the likelihood of regrowth. These findings are consistent with previous reports (5, 6, 35). This suggests that the clinical characteristics described above are useful and reliable tools for predicting the prognosis of patients with PMA.

The tumor classification involving the transcription factor or ultrastructure showed that the presence of giant lactotroph, sparsely granulated somatotroph, crooke's cell, or silent corticotroph adenomas tends to present the recurrence nature (5, 36, 37). Our study focused on the proposed IHC subtypes that are also important and potential indicators related to the progression. The study reported by Asioli et al. showed that PRL, ACHT, and FSH-LH subunits had relapse risk with high probability (20). These indices were not statistically significant when compared between the two groups in our study; it is possible that this was owing to the small sample size compared with the previous study. Although the rate of TSH adenoma was the lowest among all cases, the trend of incidence is in line with tangible clinical practice. The morbidity of this type is low based on the demographic investigation, comprising <3% of all tumors (38). We included the plurihormonal adenoma in our study. The most common type is the co-secretion of GH and PRL (39). But the combination of different hormones tends to be more complicated. Little is known with regard to the correlation between recurrence and plurihormonal tumors.

The trans-sphenoidal and endoscopic surgical methods are extensively applied to dealing with PMA (40–42). Our study did not show the correlation between operative approaches and recurrence. According to current studies, tumor size and invasive extension were decisive factors in extent of resection. The macro or giant adenomas and extensive invasion tended to be difficult to achieve grossly complete resection, although the trans-sphenoidal or endoscopic resection was used (43–45). The residual tumor is likely to be the crucial factor that affects the prognosis, suggesting that the surgical resection may be more likely to associate with intrinsic biological characteristics of tumors, compared with an operative procedure. Patients who received total resection still have the possibility of relapse (5). This indicates that surgery alone may not enable to decrease the recurrence rate. It is also important and beneficial to combine radiotherapy or other methods (46).

The consistency of adenomas probably influences the prognostic outcomes. A study showed that texture was correlated with tumor profiles, complications, and surgical resection. The hard adenomas were at higher risk of large and aggressive behavior and subtotal removal (47). This suggests that consistency is potential to predict the recurrence of PMAs. Rui et al. confirmed the utility of the radiomics method for determining the texture of PMAs (13). Future PA studies may pay more attention to the relationship between the stiffness and recurrence by radiomics.

The proliferative biomarkers of ki-67, p53, and mitosis play an important role in tumor prognosis. Although there remains the controversy of arguments, the changes of these proliferation indices are often associated with aggressive PAs. The prognoses of the patients tended to be recurrent or poorer in the presence of ki-67 ≥3, mitoses >2, and p53 overexpression based on the study by Raverot and European Society of Endocrinology (48). Above all, the most effective and useful predictive strategies are incorporating the predictors of different fields, such as clinical, imaging, and immunohistochemical examination.

## Limitations

First, the proliferations, transcription factors, ultrastructural subtypes, along with expression profiles of certain genes, were not considered in this study but may improve the performance of our classifier. Second, the pituitary scanning sequence with smaller slice thickness and interval was not applied in the study, because the protocol is not clinical routine examination; it may be considered in future studies. Third, microadenoma is another common subgroup of pituitary tumor; radiomic studies of this form of tumor are very rare. We did not include this type of tumor in the present study due to a limited sample size. Finally, this study was based in a single center and lacks external validation in multiple centers.

## Conclusion

The combination of clinicopathological characteristics and imaging is useful for predicting the recurrence of PMA within 5 years. The integrated classifier was superior to a clinical classifier and may facilitate the prediction of individualized prognosis and therapy.

## Data Availability Statement

The original contributions presented in the study are included in the article/Supplementary Material, further inquiries can be directed to the corresponding author/s.

## Ethics Statement

The studies involving human participants were reviewed and approved by the Ethics Committee of Beijing Tiantan Hospital. The Ethics Committee obtained written informed consent from the participants.

## Author Contributions

JM designed the original research. YZ conducted the research and wrote the manuscript. YZ, YLu, and XK analyzed the data. JM, TW, and YLo revised the paper. All authors contributed to the article and approved the submitted version.

## Funding

This research project was funded by National Natural Science Foundation of China (No. 61771325).

## Conflict of Interest

The authors declare that the research was conducted in the absence of any commercial or financial relationships that could be construed as a potential conflict of interest.

## Publisher's Note

All claims expressed in this article are solely those of the authors and do not necessarily represent those of their affiliated organizations, or those of the publisher, the editors and the reviewers. Any product that may be evaluated in this article, or claim that may be made by its manufacturer, is not guaranteed or endorsed by the publisher.
