# Supplementary material for: A Preoperative MRI-Based Radiomics-Clinicopathological Classifier to Predict the Recurrence of Pituitary Macroadenoma Within 5 Years
Source: Front Neurol. 2022 Jan 5;12:780628. doi: 10.3389/fneur.2021.780628 (PMC8767054; doi:10.3389/fneur.2021.780628)
Supplement: Supplementary file 1 [file Table_1.DOCX]

**SUPPLEMENTARY MATERIALS**

**Supplementary material 1.**

| **Hardy-Wilson classification** | **Knosp criteria** |
| --- | --- |
| **Grade** | **Score** |
| 1 sella normal; tumor < 10mm | 0 no extension beyond medial border |
|  | of carotid artery. Cavernous sinus  not involved |
| 2 sella enlarged; tumor ⩾ 10mm | 1. extends to but not beyond   inter-carotid line |
| 3 local perforation of sellar floor | 2 extends to lateral border of carotid |
| 4 diffuse sellar floor destruction | 3 extends beyond carotid |
| **Stage** | 4 total encasement of the  intra-cavernous carotid artery |
| 0 no suprasellar extension |  |
| A extension to suprasellar cistern |  |
| B recesses of third ventricle obliterated |  |
| C third ventricle grossly displaced |  |
| D intracranial |  |
| E into/beneath cavernous sinus |  |

From references 5 and 23

After the pre-contrast scanning was finished, the dimeglumine gadopentetate (Beilu Pharmaceutical Co., LTD) was injected into the patient with a dose of 0.2ml/kg via the antecubital venous. Once the injection was finished, the post-contrast scanning started.

**Supplementary material 2.** The parameters used for axial CE-T1 scans in each MRI scanner.

| **MRI scanner** | **TR (ms)** | **TE (ms)** | **Slice thickness (mm)** | **FOV** |
| --- | --- | --- | --- | --- |
| GE Discovery MR 750 | 2804 | 19 | 5.00 | 512×512 |
| GE Medical System Genesis Signa | 2031 | 19 | 5.00 | 512×512 |
| Siemens MAGNETOM Trio TimSystem | 2000 | 9.8 | 5.00 | 512×432 |
| Siemens MAGNETOM Verio | 1900 | 9.4 | 5.00 | 512×496 |
| Philips Ingenia | 2000 | 20 | 5.00 | 512×512 |

**Supplementary material 3.** The detailed information for all features

| **Features** | | **Numbers** |
| --- | --- | --- |
| Intensity histogram |  | 18 |
| Texture | GLDM | 14 |
|  | GLCM | 24 |
|  | GLRLM | 16 |
|  | GLSZM | 16 |
|  | NGTDM | 5 |
| Shape |  | 14 |
| Wavelet | Wavelet-HLL | 93 |
|  | Wavelet-LHL | 93 |
|  | Wavelet-LHH | 93 |
|  | Wavelet-LLH | 93 |
|  | Wavelet-HLH | 93 |
|  | Wavelet-HHH | 93 |
|  | Wavelet-HHL | 93 |
|  | Wavelet-LLL | 93 |
|  | LOG-1.5 | 93 |
|  | LOG-2.0 | 93 |
|  | LOG-2.5 | 93 |
